# Cleaning and disinfection in neonatal intensive care units: correlation of evaluation methods

**DOI:** 10.1590/0034-7167-2024-0424

**Published:** 2025-09-01

**Authors:** Maria Heloisa Silva, Aires Garcia dos Santos, André Luiz Silva Alvim, Daniel de Macedo Rocha, Verusca Soares de Souza, Alvaro Francisco Lopes de Sousa

**Affiliations:** I Universidade Federal do Mato Grosso do Sul. Três Lagoas, Mato Grosso do Sul, Brazil; II Universidade Federal de Juiz de Fora. Juiz de Fora, Minas Gerais, Brazil; III Universidade Federal do Mato Grosso do Sul. Coxim, Mato Grosso do Sul, Brazil; IV Universidade Estadual do Paraná. Paranavaí, Paraná, Brazil; V Universidade Federal do Mato Grosso do Sul. Campo Grande, Mato Grosso do Sil, Brazil; VI Hospital Sírio-Libânes, Instituto de Ensino e Pesquisa. São Paulo, São Paulo, Brazil

**Keywords:** Equipment Contamination, Disinfection, Adenosine Triphosphate, Infection Control, Cross Infection, Contaminación de Equipos, Desinfección, Adenosina Trifosfato, Control de Infecciones, Infección Hospitalaria

## Abstract

**Objectives:**

to correlate surface cleaning and disinfection monitoring methods in two neonatal intensive care units and compare threshold values for adenosine triphosphate.

**Methods:**

a correlational study with 864 assessments was conducted using adenosine triphosphate quantification, visual inspection, aerobic colony count, *Staphylococcus aureus* detection, and resistance testing before and after hygiene procedures.

**Results:**

in Unit A, correlations were found between colony count and *Staphylococcus aureus* on the counter (phase I; P=0.031) and between adenosine triphosphate and colony count in the incubator (phase II; P=0.001). In Unit B, a correlation was observed between adenosine triphosphate and colony count on the infusion pump (phase I; P=0.045). Threshold values for adenosine triphosphate were lower than 28 relative light units in Unit A and equal to or lower than 38 in Unit B.

**Conclusions:**

variations were observed in the correlations and threshold values for adenosine triphosphate between the analyzed units.

## RESUMEN

**Objetivos:**

correlacionar los métodos de monitoreo de la limpieza y desinfección de superficies en dos unidades de cuidados intensivos neonatales y comparar los valores de corte del trifosfato de adenosina.

**Métodos:**

estudio correlacional con 864 evaluaciones, utilizando cuantificación de trifosfato de adenosina, inspección visual, recuento de colonias de bacterias aerobias, detección de *Staphylococcus aureus* y pruebas de resistencia, antes y después de los procedimientos de higiene.

**Resultados:**

en la Unidad A se observó correlación entre el recuento de colonias y *Staphylococcus aureus* en el mesón (fase I; P=0,031), y entre trifosfato de adenosina y colonias en la incubadora (fase II; P=0,001). En la Unidad B, se identificó correlación entre trifosfato de adenosina y colonias en la bomba de infusión (fase I; P=0,045). Los valores de corte del trifosfato de adenosina fueron inferiores a 28 unidades de luz en la Unidad A y menores o iguales a 38 en la Unidad B.

**Conclusiones:**

se observaron variaciones en las correlaciones y en los valores de corte entre las unidades analizadas.

## INTRODUCTION

Multidrug-resistant microorganisms frequently contaminate surfaces in healthcare settings^(1)^. Among these pathogens are *Clostridioides difficile*, vancomycin-resistant enterococci (VRE), methicillin-resistant *Staphylococcus aureus* (MRSA), *Acinetobacter baumannii*, *Pseudomonas aeruginosa*, and noroviruses. These microorganisms possess an intrinsic ability to survive for extended periods on surfaces^(2,3)^.

To minimize the occurrence of healthcare-associated infections (HAIs), one strategy that can be employed is surface cleaning and disinfection (SCD), a measure that extends beyond mere aesthetic considerations^(4)^. Proper SCD in hospital equipment and environments is essential to prevent the spread of pathogens and reduce the risk of cross-infections among patients^(1,5)^.

While most healthcare facilities have teams responsible for SCD, the use of quantitative and qualitative methods to monitor the effectiveness of this process on environmental surfaces remains limited^(6)^. In this context, various SCD monitoring methods are available, including adenosine triphosphate (ATP) assays, total aerobic counts, visual inspection, and microbiological identification, such as detecting *Staphylococcus aureus* and its methicillin resistance, among others. It is important to note that each method has its own advantages and disadvantages^(7)^.

ATP measurement offers a simple, rapid, and quantitative approach to assessing the organic matter load on surfaces. However, concerns have been raised regarding the lack of a standardized reference value and the difficulty in correlating ATP levels with bacterial counts due to the influence of disinfectant residues on environmental surfaces^(1,8)^.

Another method, total aerobic microorganism counts expressed as colony-forming units (CFU) per cm², involves small Petri dishes containing agar that are directly applied to flat surfaces. These plates provide the advantage of quantitatively measuring the microbial load on surfaces. Despite its efficacy, this method has significant drawbacks. First, it entails high operational costs, as it requires specialized laboratory equipment. Additionally, the sample processing time is relatively long, with results typically available 48 hours after collection. These factors may limit its applicability in situations requiring rapid responses or in budget-constrained contexts^(9-11)^.

Visual inspection is the most commonly used method to evaluate the effectiveness of SCD practices, allowing for a direct analysis of the removal of visible debris, dust, and moisture. However, this method has limitations, as it does not assess microbial contamination or the presence of organic matter. Moreover, its subjectivity can vary depending on the evaluator, making it unable to detect microorganisms. Nonetheless, visual inspection can be useful for monitoring adherence to SCD protocols, although it is important to incorporate other evaluation methods to ensure effective contamination reduction in hospital environments^(12,13)^.

The identification of microorganisms present on surfaces is of great importance and should be incorporated as a monitoring method. For example, among the microorganisms of epidemiological relevance is *Staphylococcus aureus*, one of the primary human pathogens, known for its wide variety of virulence factors and its ability to develop resistance to multiple antibiotics, resulting in the continuous emergence of new clones. MRSA is one of the most common resistance phenotypes due to the widespread use of methicillin in clinical practice and is also a nosocomial pathogen associated with healthcare settings^(14,15)^.

Recognizing the implications of ineffective SCD and the limitations of monitoring methods, the combined use of multiple methods becomes essential^(7,16)^. Although the literature includes individual studies evaluating the correlation between monitoring methods in various settings—such as outpatient clinics, pediatrics, urgent care units, nursing homes, hospital emergency services, and primary care^(13,16)^—there remains a significant research gap specific to neonatal intensive care units (NICUs).

In this environment, due to the vulnerability of patients and the need for strict microbial load control, it is essential to investigate the correlation between specific monitoring methods and establish appropriate ATP threshold values, considering the unique characteristics of the neonatal setting. This investigation is relevant because it contributes to the establishment of more precise and effective parameters for SCD in NICUs, minimizing the risk of infections and enhancing patient safety.

Given this context, the study aims to address two knowledge gaps: Is there a correlation between SCD monitoring methods in two NICUs (located in the same city, with the same number of beds, and following the same SCD protocol)? Is the ATP threshold value similar between the two NICUs? The hypothesis of this study is that there is a variable correlation between SCD monitoring methods in the two NICUs, despite both being located in the same city, having the same number of beds, and utilizing similar SCD protocols.

## OBJECTIVES

To correlate SCD monitoring methods in two NICUs and compare ATP threshold values.

## METHODS

### Ethical Aspects

This research adhered to all national and international ethical guidelines and received approval from the Research Ethics Committee for Human Subjects at the Federal University of Mato Grosso do Sul. Informed consent (ICF) was obtained online from all individuals involved in the study.

### Study Design

This is a cross-sectional, analytical, and comparative study conducted between June and November 2023, following the recommendations of the STROBE checklist.

### Population and Sample

Samples were collected from two NICUs in the Central-West region of Brazil. Both NICUs have ten beds and multidisciplinary teams. NICU A (private) was staffed by 23 nursing professionals (NPs) and 2 environmental services professionals (ESPs), while NICU B (public) had 28 NPs and 4 ESPs. Both units serve as referral centers for a population of 132,152 inhabitants.

### Study Protocol

Using an intentional and non-probabilistic approach, the following items were selected for analysis: counters (marble in NICU A and wood in NICU B), incubators (metal and acrylic structures in both units), chairs (polyvinyl chloride and polyester mesh), infusion pumps (polycarbonate in both units), tables (stainless steel in both units), and scales (polypropylene in both units).

The selection of surfaces was based on systematic observation, focusing on high-touch surfaces^(12,13,16)^. The criteria for selecting surfaces considered not only the frequency of contact but also the material composition, as this can significantly influence microbial adhesion and ATP evaluation. Materials like polycarbonate and stainless steel have relatively smooth surfaces, which may hinder microorganism adhesion; conversely, porous surfaces like wood or fabric can promote greater microbial adherence. These differences are crucial for accurate ATP evaluation, as the amount of retained and detected organic matter can vary based on the material’s characteristics^(12)^.

Sample collections were performed twice a week, with six samples collected before and six after the cleaning process, totaling 12 daily and 24 weekly samples. This sampling strategy allows for consistent analysis of microbial load at different time points, capturing the effectiveness of the SCD process in the NICUs over time. By the end of each month, 96 samples were collected per method, as shown in Chart 1.

**Chart 1:** Number of evaluations performed by method in each study phase, Três Lagoas, Mato Grosso do Sul, Brazil, 2023 (N=864)

Method	Phase I (30 days)	Phase II (30 days)	Phase III (30 days)	Total Assessments
**Visual Inspection**		96	96	288
**ATP**	96	96	96	288
**CFU**	96	96	96	288
***Staphylococcus aureus* / MRSA**	96	96	96	288
**Total**	288	288	288	864

*ATP – adenosine triphosphate; CFU – colony-forming units; MRSA – methicillin-resistant Staphylococcus aureus*.

Both institutions had protocols for concurrent and terminal cleaning, with terminal cleaning performed once a week or whenever a patient was discharged, transferred, or deceased. Concurrent cleaning was performed once a day. NPs and ESPs were responsible for SCD.

However, in both NICUs, it was observed that each professional used different products and materials for performing SCD, indicating a lack of standardization prior to the educational intervention conducted in phase II of the study. No specific training on SCD was provided in either NICU before the study; training sessions were limited to topics related to handwashing techniques and the use of standard precautions.

### Data Collection and Organization

The monitoring methods used included visual inspection, ATP bioluminescence, CFU counting, and the identification of pathogenic microorganisms such as MRSA. Data collection was conducted by the researcher immediately before and 5 minutes after completing the morning or afternoon SCD. This procedure ensured that surfaces were completely dry to avoid potential interference in readings due to contact with sanitizing products^(17)^.

The study consisted of three phases: I - situational diagnosis, II - educational intervention and immediate monitoring, and III - short-term monitoring. After completing the evaluations in the hospitals, an educational intervention was conducted, presenting the results and proposing improved SCD procedures. The educational intervention was initially evaluated by three members of the Hospital Infection Control Committee (HICC) from both institutions, comprising an infectious disease physician and two nurses. After validation by the HICC, the intervention was coordinated with the NICU nursing teams and environmental services teams.

It was standardized that SCD would be performed twice per shift (at the beginning and end of each shift). Considering that surfaces can become recolonized over time and ATP values can increase, intermediate cleaning twice daily showed benefits compared to cleaning performed only once per day^(18)^.

The use of a hospital-grade disinfectant for the six monitored surfaces was also standardized. The disinfectant was based on alkyl dimethyl benzyl ammonium chloride, recommended for the disinfection of fixed surfaces and non-critical articles due to its microbicidal action and broad-spectrum activity against bacteria, yeasts, fungi, viruses, and spores. The product should be applied with the surface left wet for 10 minutes^(11)^. The use of microfiber cloths was also established for cleaning, given their superior performance in microbial removal^(19)^.

To measure organic matter, ATP bioluminescence was performed using a portable luminometer (NGi 3M™ Clean-Trace™, St. Paul, MN) and a swab (3M™ Clean-Trace™ ATP Surface). Surfaces with readings below 250 RLU were considered clean^(20,21)^. The sampling area for ATP swab collection was standardized at 25 cm²^(22)^.

To monitor total aerobic microorganisms, 24 cm² contact plates of the Rodac Plate^®^ type (*Biocen do Brasil*), containing tryptic soy agar and neutralizers, were used. After pressing the plates onto the surfaces for 10 seconds, they were incubated at 37°C for 24 to 48 hours. The plates were analyzed using a digital colony counter (Logen LS6000; Texas Instruments Inc., Dallas, TX), with clean surfaces defined as those with less than 2.5 CFU/cm², corresponding to fewer than 60 CFU on a 24 cm² plate.

Additionally, the presence of *Staphylococcus aureus* was analyzed using Petrifilm™ plates (3M™, St. Paul, MN, USA), a ready-to-use culture medium system containing a cold-water-soluble gelling agent. The modified Baird Parker chromogenic medium on the plate is selective and differential for *Staphylococcus aureus*. Colonies with a reddish-violet color confirmed the presence of *Staphylococcus aureus*. The plates were inoculated at 35°C for 24 to 48 hours. Colonies with a reddish-violet color were then subcultured onto 90 x 15 mm Chromagar MRSA plates, which feature a chromogenic medium designed for isolating and differentiating MRSA. After 48 hours, plates that displayed color changes indicated the presence of MRSA.

### Visual Surface Assessment

For visual surface assessment, an initial inspection was conducted to identify areas of dirt, stains, or any type of visible residue. Pre-established criteria were used to evaluate cleanliness, focusing on the removal of visible dirt, dust, and other contaminants^(23)^.

### Data Analysis

The data were analyzed using the following statistical tests: a two-proportion test to compare the frequency of failed surfaces between monitoring methods (CFU, ATP, and *Staphylococcus aureus*); Spearman’s correlation to examine potential correlations between the quantification of continuous variables (ATP and microbial counts on each surface before and after cleaning and disinfection); and ROC curve analysis to determine whether ATP bioluminescence testing is effective for assessing the quality of cleaning and disinfection relative to the microbiological gold standard.

All statistical tests were applied with a significance level of 5% (P < 0.05), and the software used included Minitab 17 (Minitab Inc.) and MedCalc 16.8 (MedCalc^®^).

## RESULTS

At the end of the three phases, 864 evaluations were conducted, including ATP measurements, visual inspection, CFU counting, *Staphylococcus aureus* (SA) detection, and resistance testing. The data are presented for the private NICU A and the public NICU B.

Table 1 presents the Spearman correlation coefficients and their corresponding P-values for the methodologies employed in the study for NICU A.

**Table 1:** Spearman correlation coefficient (*p* value) between adenosine triphosphate (relative light units) and aerobic bacteria (colony-forming units) in samples collected from surfaces – Neonatal Intensive Care Unit A

Phase	Surfaces	ATP and CFU		ATP and SA		CFU and SA	
			*p* value^2^	r	*p* value	r	*p* value
I	Incubator	-0.098	0.818	0.252	0.547	0.000	1.000
	Preparation Table	0.139	0.742	0.427	0.291	0.263	0.529
	Infusion Pump	-0.643	0.086	0.206	0.624	-0.289	0.488
	Scale	-0.012	0.978	0.412	0.310	0.415	0.307
	Chair	-0.171	0.686	-0.084	0.844	-0.049	0.908
	Counter	0.587	0.126	0.179	0.672	0.754	0.031
II	Incubator	0.922	0.001	0.234	0.577	0.486	0.222
	Preparation Table	0.299	0.471	-0.577	0.134	0.249	0.552
	Infusion Pump	0.527	0.180	0.655	0.078	-0.094	0.825
	Scale	0.415	0.307	0.183	0.664	0.217	0.606
	Chair	0.311	0.453	0.430	0.288	0.117	0.782
	Counter	-0.024	0.955	-0.192	0.650	0.217	0.606
III	Incubator	-0.572	0.138	-	-	-	-
	Preparation Table	0.455	0.257	-	-	-	-
	Infusion Pump	-0.275	0.509	0.083	0.845	0.412	0.310
	Scale	-0.482	0.227	-0.218	0.604	0.331	0.423
	Chair	0.530	0.177	-0.082	0.846	0.000	1.000
	Counter	-0.610	0.108	-	-	-	-

*ATP – adenosine triphosphate; CFU – colony-forming units; SA – Staphylococcus aureus*;

1 *r: Spearman’s correlation coefficient;*

2 *p value: Refers to the Spearman correlation test with p<0.05*.

**Figure 1:**
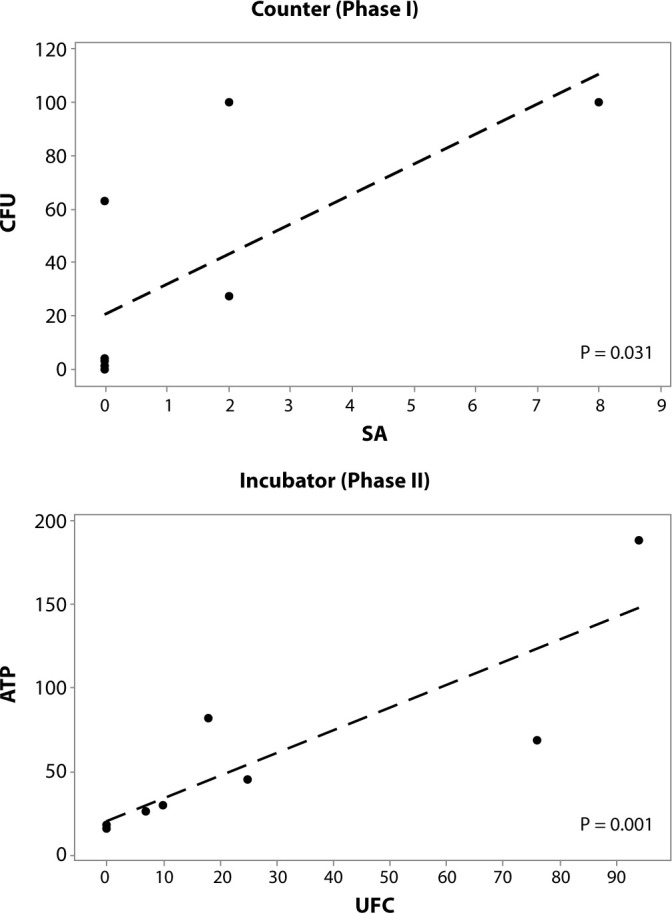
Correlation between aerobic bacteria (colony-forming units) and Staphylococcus aureus on the counter (phase I), and between adenosine triphosphate (relative light units) and aerobic bacteria in the incubator (phase II) – Neonatal Intensive Care Unit A

The results revealed specific correlations across different surfaces and evaluation phases. In phase I, a significant positive correlation was observed between CFU and SA quantification on the counter (P = 0.031), indicating that higher CFU values are associated with higher SA values. In phase II, a significant positive correlation was also identified between ATP and CFU quantification in the incubator (P = 0.001), suggesting that as ATP values increase, CFU values also increase. These correlations are visually presented in Figure 1.

Table 2 provides the application of Spearman’s correlation test using data from NICU B.

**Table 2:** Spearman correlation coefficient (*p* value) between adenosine triphosphate (relative light units) and aerobic bacteria (colony-forming units) in samples collected from surfaces – Neonatal Intensive Care Unit B

Phase	Surfaces	ATP and CFU		ATP and SA		CFU and SA	
		r^1^	*p* value^2^	r	*p* value	r	*p* value
I	Incubator	0.214	0.610	-0.247	0.555	0.289	0.488
	Preparation Table	0.482	0.227	-0.325	0.432	0.342	0.407
	Infusion Pump	-0.719	0.045	0.701	0.053	-0.390	0.339
	Scale	0.548	0.160	-	-	-	-
	Chair	-0.036	0.932	0.342	0.408	-0.210	0.618
	Counter	0.252	0.548	0.055	0.898	0.590	0.124
II	Incubator	-0.575	0.136	0.038	0.929	0.147	0.729
	Preparation Table	0.190	0.651	0.507	0.199	0.114	0.788
	Infusion Pump	0.168	0.691	-0.051	0.905	-0.223	0.595
	Scale	-0.310	0.456	-	-	-	-
	Chair	0.216	0.608	-	-	-	-
	Counter	0.168	0.691	-	-	-	-
III	Incubator	0.275	0.509	0.082	0.846	0.083	0.845
	Preparation Table	0.434	0.283	0.327	0.429	0.237	0.573
	Infusion Pump	0.072	0.866	-	-	-	-
	Scale	0.262	0.531	-	-	-	-
	Chair	0.476	0.233	0.203	0.630	0.343	0.406
	Counter	0.071	0.867	0.577	0.134	0.247	0.555

*ATP – adenosine triphosphate; CFU – colony-forming units; SA – Staphylococcus aureus;*

1 *r: Spearman’s correlation coefficient;*

2 *p value: Refers to the Spearman correlation test with p<0.05*.

**Figure 2:**
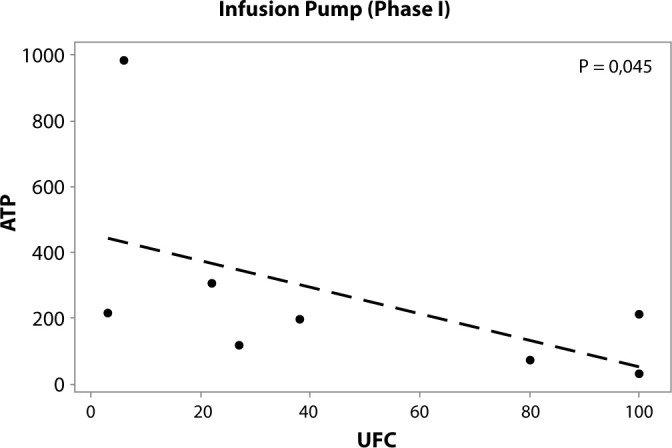
Correlation between adenosine triphosphate (relative light units) and aerobic bacteria (colony-forming units) on the infusion pump (phase I)

The data presented in Table 2 show a significant correlation between ATP and CFU quantification on the infusion pump during phase I (P = 0.045). Notably, this correlation is negative, indicating that an increase in microbial count is associated with a decrease in the ATP score (Figure 2).

**Figure 3:**
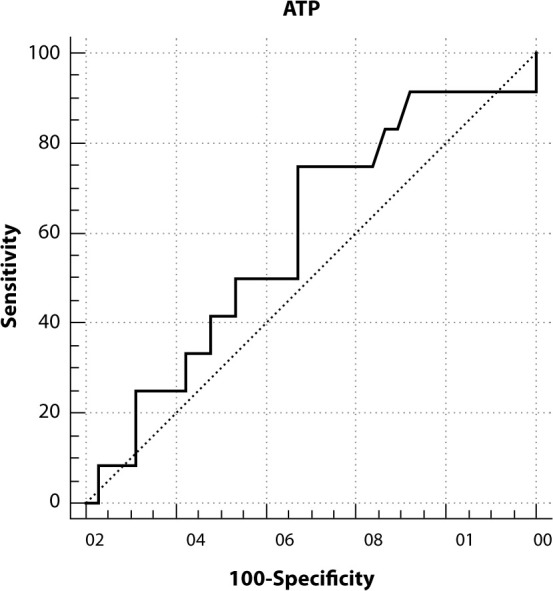
Receiver operating characteristic curve for adenosine triphosphate quantification methods in relation to the microbial count gold standard

The ROC curve analysis for the data from NICU A enabled the evaluation of ATP quantification and visual inspection methods compared to the microbiological gold standard. ATP quantification via bioluminescence demonstrated a sensitivity of 75%, specificity of 52.8%, a positive predictive value of 61.37%, and a negative predictive value of 67.86%. Based on the reference of CFU counts < 2.5 CFU/cm² to classify a surface as clean, the ROC analysis indicates that surfaces with ATP readings below 28 URL can be considered approved. This suggests an effective criterion for assessing surface cleanliness in NICU A. This value represents the point of highest specificity and sensitivity, as illustrated in the figure below.

**Figure 4:**
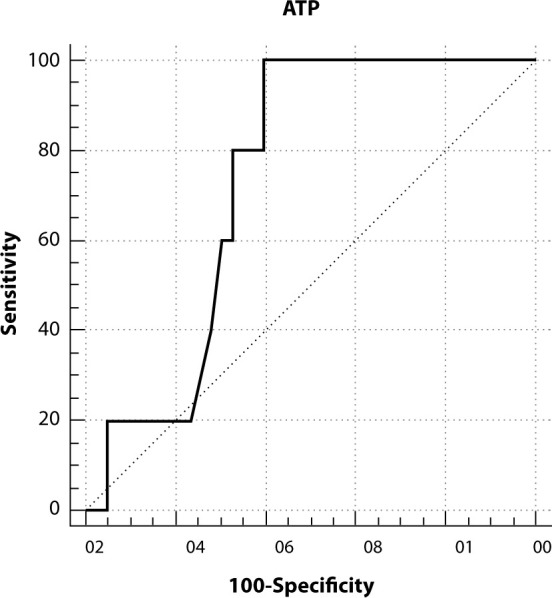
Receiver operating characteristic curve for adenosine triphosphate quantification methods in relation to the microbial count gold standard

The ROC curve analysis using data from NICU B, evaluating ATP quantification via bioluminescence in comparison to microbial counts, revealed the following results: sensitivity of 100%, specificity of 60.5%, a positive predictive value of 71.68%, and a negative predictive value of 100%. Based on the reference of CFU counts < 2.5 CFU/cm² to define a clean surface, the ROC analysis suggests that surfaces with ATP readings less than or equal to 38 URL can be considered approved. This cutoff point was identified as the one with the highest specificity and sensitivity, as illustrated in Figure 4.

## DISCUSSION

The study aimed to correlate SCD monitoring methods in two NICUs and compare ATP threshold values. The analysis revealed significant differences in the correlation between monitoring methods and the threshold values of the first test between the two units, despite their use of similar disinfection protocols and products.

The correlation between ATP, CFU, and visual inspection on NICU surfaces holds significant clinical relevance for controlling HAIs. The identification of positive correlations between ATP and CFU on specific surfaces suggests that ATP can be a useful tool for assessing the presence of organic matter and potentially indicating microbiological contamination. However, since ATP quantification reflects total biological matter and not exclusively microorganisms, isolated interpretations may lead to errors in evaluating cleaning effectiveness.

Visual inspection, while important, demonstrated limitations when used in isolation, as visually clean surfaces can still show high ATP and CFU levels, indicating the presence of microorganisms and a potential infection risk. Thus, the correlation between these measurements highlights the need for a multidimensional approach, combining different monitoring methods to ensure safer environments for patients, particularly in NICUs, where newborns are highly vulnerable to infections^(13,23-25)^.

Regarding method correlation, it was observed that, even though the two NICUs are located in the same city, follow similar cleaning protocols, have the same number of beds, and use the same products for SCD, they displayed differing data regarding method correlation and ATP threshold values. In NICU A, a positive correlation was observed only between CFU and *Staphylococcus aureus* quantification on the counter in phase I (P = 0.031) and between ATP and CFU quantification in the incubator in phase II (P = 0.001). In contrast, in NICU B, a correlation was found between ATP and CFU quantification on only one surface—the infusion pump in phase I (P = 0.045).

This variation has also been observed in studies conducted in different scenarios. In a pediatric inpatient ward, a significant correlation was identified between ATP measurements and microbial counts on the bed rail (P = 0.009) and the chair (P = 0.018)^(16)^. In a study conducted in an emergency department, a significant correlation was also found between ATP quantification and microbial counts on the handle of the women’s restroom door (P = 0.526; P = 0.008)^(26)^.

In research conducted in primary care, a significant correlation was observed between ATP quantification and aerobic microbial counts only on the patient examination table (P = 0.001), with a Spearman correlation coefficient of r = 0.649^(27)^. In another study conducted on 10 hospital and nursing home surfaces, a weak correlation was observed between ATP (URL) values and CFU counts (R = 0.244; P < 0.001)^(28)^.

Variations in correlation results are influenced by several factors, including the epidemiological profile of colonization/environmental contamination and the fact that the correlation between ATP and CFU is not always linear. A surface with a high organic matter load identified by ATP does not necessarily have a high microbial contamination load, as measured by CFU^(16)^. In this context, it is important to emphasize that ATP quantifies a wide range of biological material, with colonies being just one of many elements that may be present in organic matter. Thus, ATP does not exclusively indicate the microbial load present on a surface^(22)^.

When comparing ROC curve parameters, differences were observed in sensitivity, specificity, positive predictive value, and negative predictive value between the two NICUs. These variations in sensitivity and specificity values, when comparing the two NICUs, are also noted in other individual studies conducted in various settings (primary care, outpatient clinics, and emergency units) that evaluated ATP quantification and visual inspection using microbial count as the gold standard in the ROC curve^(12,13,23)^. These findings underscore the importance of employing multiple methods to monitor SCD due to their differing sensitivities and specificities^(26)^.

Regarding ATP threshold values, in the private NICU, the value was below 28 URL, while in the public NICU, it was less than or equal to 38 URL. Previous studies have identified different ATP threshold values, such as 48 URL in primary care and 49 URL in outpatient care^(13,27)^. The variation in these values may be related to differences in ATP threshold criteria and variability in the systems used to monitor ATP. Additionally, failures in soil removal can allow ATP to persist on surfaces for more than 24 hours, even after microorganisms have been killed by disinfectants, leading to elevated ATP results and low CFU counts^(23)^. In other words, microorganisms are only one component of organic matter^(28)^.

Following effective cleaning and disinfection protocols, such as using bleach-based products, quaternary ammonium disinfectants, and liquid or vaporized hydrogen peroxide, enables environmental services professionals to eliminate or reduce the presence of pathogens in healthcare environments. This is particularly crucial for controlling the spread of pathogens that pose a serious threat to patient safety^(5)^.

It is worth noting that various types of devices and brands are available for ATP measurement, which also contribute to result variability^(22)^. Another relevant aspect concerns the sampling area used for ATP swab collection. If ATP readings are not calibrated to the sampling area, the resulting data may lead to complex interpretations and make comparisons challenging, particularly on non-uniform surfaces^(22)^.

In a study conducted in three phases, the authors observed that ATP values increased five hours after cleaning in phases 1 and 2 and eight hours after cleaning in phase 3. Additionally, older, structurally outdated wards showed higher ATP levels^(18)^. Although ATP should not replace microbiological tests, it provides immediate feedback to professionals regarding the SCD process being performed^(29)^.

To minimize ATP variability, the study implemented duplicate sampling using swabs. However, the same study identified a false-negative rate of only 10%, referring to cases where the first sample showed an approval result (< 100 URL), while the second sample failed (> 100 URL)^(30)^.

### Study limitations

The ROC curve analysis for *Staphylococcus aureus* quantification could not be performed due to insufficient sample representativeness and the high frequency of null values. Although the Hawthorne effect—where professionals alter their behavior when they know they are being observed—was a potential concern, it was minimized. In phase 1, the professionals were not informed of the study’s objectives, and in subsequent phases, sample collection was conducted before the arrival of ESPs in the unit, ensuring that SCD practices in the NICU were not influenced by observation.

It is important to highlight that the lack of standardization in cleaning products and the absence of training for teams responsible for SCD are critical issues. These factors may contribute to variations in results and increase the likelihood of bias, as each professional’s technique can impact the effectiveness of SCD.

### Contributions to the Field

While the importance of SCD monitoring methods is widely recognized, visual inspection remains the predominant method in most services, often focusing primarily on aesthetic aspects. This study underscores the need to incorporate other evaluation methods. Furthermore, it addresses a gap in the literature regarding the effectiveness of monitoring methods in neonatal ICUs, emphasizing the relevance of a multifaceted approach tailored to the specificities of each environment when establishing SCD standards.

Implementing educational interventions and standardizing procedures can improve disinfection efficacy and, consequently, reduce the risk of hospital-acquired infections, ensuring a safer environment for neonatal patients.

## CONCLUSIONS

This study revealed significant variations in the correlation of SCD monitoring methods between the two NICUs analyzed, despite both being located in the same city, having the same number of beds, and using similar disinfection protocols and products. In NICU A, positive correlations were observed between CFU and *Staphylococcus aureus* and between ATP and CFU, whereas in NICU B, no positive correlations were identified among the methods. Additionally, ATP threshold values differed between the NICUs, indicating varying levels of disinfection efficacy.

These findings highlight the need for customized approaches to cleaning and disinfection monitoring, taking into account the specificities of each unit. The divergence in results suggests that factors unique to each NICU, such as cleaning practices, surface characteristics, and healthcare professionals’ behavior, can influence the effectiveness of disinfection methods.

The variation in ATP threshold values also underscores the importance of defining specific standards for each context rather than applying a universal value. Therefore, it is recommended to adapt cleaning monitoring protocols to the unique characteristics of each unit and adjust ATP threshold values based on local conditions to optimize disinfection processes and ensure patient safety.

## Data Availability

Not applicable.
