# Supplementary material for: Cleaning and disinfection in neonatal intensive care units: correlation of evaluation methods
Source: Rev Bras Enferm. 2025 Sep 1;78(3):e20240424. doi: 10.1590/0034-7167-2024-0424 (PMC12408087; doi:10.1590/0034-7167-2024-0424)
Supplement: Translation

# Translation: Limpeza e desinfecção em unidade de terapia intensiva neonatal: correlação de métodos de avaliação

**DOI:** 10.1590/0034-7167-2024-0424pt

**Authors:** Maria Heloisa Silva, Aires Garcia dos Santos, André Luiz Silva Alvim, Daniel de Macedo Rocha, Verusca Soares de Souza, Alvaro Francisco Lopes de Sousa

**Affiliations:** I Universidade Federal do Mato Grosso do Sul. Três Lagoas, Mato Grosso do Sul, Brazil; II Universidade Federal de Juiz de Fora. Juiz de Fora, Minas Gerais, Brazil; III Universidade Federal do Mato Grosso do Sul. Coxim, Mato Grosso do Sul, Brazil; IV Universidade Estadual do Paraná. Paranavaí, Paraná, Brazil; V Universidade Federal do Mato Grosso do Sul. Campo Grande, Mato Grosso do Sil, Brazil; VI Hospital Sírio-Libânes, Instituto de Ensino e Pesquisa. São Paulo, São Paulo, Brazil

**Keywords:** Desinfecção, Trifosfato de Adenosina, Controle de Infecções, Infecção Hospitalar, Contaminação de Equipamentos

## Abstract

**Objetivos:**

correlacionar métodos de monitoramento da limpeza e desinfecção de superfícies em duas unidades de terapia intensiva neonatal e comparar valores de corte do trifosfato de adenosina.

**Métodos:**

estudo correlacional com 864 avaliações, utilizando quantificação de trifosfato de adenosina, inspeção visual, contagem de colônias de bactérias aeróbias, detecção de *Staphylococcus aureus* e testagem de resistência, antes e após os procedimentos de higienização.

**Resultados:**

na unidade A, houve correlação entre colônias e *Staphylococcus aureus* no balcão (fase I; P=0,031) e entre trifosfato de adenosina e colônias na incubadora (fase II; p=0,001). Na unidade B, identificou-se correlação entre trifosfato de adenosina e colônias na bomba de infusão (fase I; p=0,045). Os valores de corte do trifosfato de adenosina foram inferiores a 28 unidades de luz na unidade A e iguais ou inferiores a 38 na unidade B.

**Conclusões:**

observou-se variação nas correlações e nos valores de corte entre as unidades analisadas.

## INTRODUÇÃO

Microrganismos multirresistentes frequentemente contaminam superfícies dos ambientes de assistência à saúde^(1)^. Entre esses patógenos estão *Clostridioides difficile*, enterococos resistentes à vancomicina (VRE), *Staphylococcus aureus* resistente à meticilina (MRSA), *Acinetobacter baumannii*, *Pseudomonas aeruginosa* e Norovírus. Esses microrganismos possuem uma capacidade intrínseca de sobrevivência prolongada em superfícies^(2,3)^.

Visando minimizar a ocorrência das Infecções Relacionadas à Assistência à Saúde (IRAS), uma das estratégias que pode ser utilizada corresponde à Limpeza e Desinfecção de Superfícies (LDS), medida que vai além da questão estética^(4)^. A realização adequada da LDS em equipamentos e ambientes hospitalares é fundamental para evitar a disseminação de patógenos e reduzir o risco de infecções cruzadas nos pacientes^(1,5)^.

Embora se observe que a maioria dos serviços de saúde possui equipes responsáveis pelo processo de LDS, ainda são limitados os que utilizam métodos quanti-qualitativos para monitorar a eficácia desse processo em superfícies ambientais^(6)^. Nesse sentido, existem diversas modalidades de monitoramento da LDS, entre as quais se incluem: o ensaio com trifosfato de adenosina (ATP), contagem de aeróbios totais, inspeção visual e identificação microbiológica, como o *Staphylococcus aureus* e sua resistência à meticilina, entre outros. É importante destacar que cada método possui vantagens e desvantagens^(7)^.

A mensuração de ATP oferece uma abordagem simples, rápida e quantitativa para avaliar a carga de matéria orgânica presente na superfície. No entanto, preocupações têm sido levantadas, incluindo a falta de um valor de referência padronizado e a dificuldade em correlacionar os níveis de ATP com as contagens bacterianas, devido à influência de resíduos de desinfetantes nas superfícies ambientais^(1,8)^.

Outro método, a contagem de microrganismos aeróbios totais expressos em Unidades Formadoras de Colônias (UFC) por cm², utiliza pequenas placas de Petri contendo ágar, que são aplicadas diretamente nas superfícies planas. Essas placas oferecem a vantagem de mensurar a carga microbiana existente na superfície de maneira quantitativa. Apesar de sua eficácia, esse método apresenta algumas desvantagens significativas. Primeiramente, envolve um alto custo operacional, uma vez que exige a utilização de equipamentos laboratoriais especializados. Além disso, o tempo de processamento das amostras é relativamente longo, com resultados disponíveis, em média, 48 horas após a coleta. Esses fatores podem limitar sua aplicabilidade em situações que requerem respostas rápidas ou em contextos com restrições orçamentárias^(9-11)^.

A inspeção visual é o método mais comumente utilizado para avaliar a eficácia das práticas de LDS, permitindo uma análise direta da remoção de detritos visíveis, poeira e umidade. No entanto, este método apresenta limitações, pois não proporciona uma avaliação da contaminação microbiana nem da presença de matéria orgânica. Além disso, sua subjetividade pode variar de acordo com o avaliador, não sendo capaz de detectar a presença de microrganismos. Apesar disso, a inspeção visual pode ser útil para monitorar a adesão aos protocolos de LDS, embora seja importante considerar outras formas de avaliação para garantir a eficácia na redução da contaminação em ambientes hospitalares^(12,13)^.

A identificação dos microrganismos presentes nas superfícies é de grande importância e deve ser incorporada como método de monitoramento. Por exemplo, entre os microrganismos de relevância epidemiológica encontra-se o *Staphylococcus aureus*, um dos principais patógenos humanos, conhecido por sua ampla variedade de fatores de virulência e pela capacidade de desenvolver resistência a múltiplos antibióticos, resultando no surgimento contínuo de novos clones. O MRSA é um dos fenótipos mais comuns de resistência, devido ao uso generalizado de meticilina na prática clínica, além de ser um patógeno nosocomial associado aos ambientes de saúde^(14,15)^.

Reconhecendo as implicações de uma LDS ineficaz e as limitações dos métodos de monitoramento, torna-se importante o uso combinado de métodos^(7,16)^. Embora a literatura apresente estudos individuais que avaliaram a correlação entre métodos de monitoramento em diferentes cenários, como clínicas ambulatoriais, pediatria, unidades de pronto atendimento, lares de idosos, serviços hospitalares de emergência e atenção primária^(13,16)^, ainda existe uma lacuna de pesquisa no contexto específico das Unidades de Terapia Intensiva Neonatal (UTIN).

Nesse ambiente, devido à vulnerabilidade dos pacientes e à necessidade de controle rigoroso da carga microbiana, é fundamental investigar a correlação entre métodos específicos de monitoramento e definir valores de corte adequados para ATP, considerando as particularidades do ambiente neonatal. Essa investigação é relevante porque contribui para estabelecer parâmetros mais precisos e eficazes para a LDS em UTIN, minimizando os riscos de infecções e aumentando a segurança dos pacientes.

Frente ao exposto, este estudo visa responder a duas lacunas de conhecimento: existe correlação entre os métodos de monitoramento da LDS em duas unidades de UTIN (localizadas na mesma cidade, com o mesmo número de leitos e o mesmo protocolo de LDS)? O ponto de corte para o ATP entre as duas UTIN é semelhante? A hipótese deste estudo é que existe uma correlação variável entre os métodos de monitoramento da LDS nas duas unidades de UTIN, apesar de ambas estarem localizadas na mesma cidade, com o mesmo número de leitos e utilizarem protocolos semelhantes de LDS.

## OBJETIVOS

Correlacionar os métodos de monitoramento da LDS em duas UTIN e comparar o valor de corte para o ATP.

## MÉTODOS

### Aspectos éticos

Esta pesquisa seguiu todas as diretrizes éticas nacionais e internacionais e recebeu aprovação do Comitê de Ética em Pesquisa com Seres Humanos da Universidade Federal de Mato Grosso do Sul. O Consentimento Livre e Esclarecido foi obtido de todos os indivíduos envolvidos no estudo por meio online.

### Tipo de estudo

Trata-se de um estudo do tipo transversal, analítico e comparativo, seguindo as recomendações do checklist STROBE, realizado entre os meses de junho e novembro de 2023.

### População e amostra

As amostras foram obtidas em duas UTIN na região Centro-Oeste do Brasil. Ambas possuem dez leitos e contam com equipe multiprofissional. A UTIN A (privada) era composta por 23 profissionais de enfermagem (PE), além de 2 profissionais dos serviços ambientais (PSA). Já a UTIN B (pública) contava com 28 PE e 4 PSA. Ambas as unidades são referência para uma população de 132.152 habitantes.

### Protocolo do estudo

Adotando uma abordagem intencional e não probabilística, foram elencados os seguintes itens para análise: balcão (mármore na unidade A e madeira na unidade B), incubadora (estrutura metálica e acrílica em ambas as unidades), poltrona (policloreto de vinila e malha de poliéster), bomba de infusão (policarbonato em ambas as unidades), mesa (aço inoxidável em ambas as unidades) e balança (polipropileno em ambas as unidades).

A seleção das superfícies foi baseada em observação sistemática, com foco em superfícies de alta frequência de toque^(12,13,16)^. O critério de escolha considerou não apenas a frequência de contato, mas também a composição dos materiais, uma vez que esta pode influenciar significativamente a aderência microbiana e a avaliação de ATP. Materiais como policarbonato e aço inoxidável apresentam superfícies relativamente lisas, o que pode dificultar a fixação de microrganismos; por outro lado, superfícies de madeira ou tecido, devido à sua porosidade, podem favorecer maior aderência microbiana. Essas diferenças são essenciais para uma avaliação precisa de ATP, já que a quantidade de matéria orgânica retida e detectada pode variar conforme as características do material^(12)^.

As coletas de amostras ocorreram duas vezes por semana, com seis amostras coletadas antes e seis após o processo de limpeza, totalizando 12 amostras diárias e 24 amostras semanais. Essa representatividade permite uma análise consistente da carga microbiana em diferentes momentos, capturando a eficácia do processo de LDS nas UTIN ao longo do tempo. Dessa forma, ao final de cada mês, foram realizadas 96 coletas por método, conforme ilustrado no Quadro 1.

**Quadro 1:** Quantidade de avaliações realizadas por método em cada etapa do estudo, Três Lagoas, Mato Grosso do Sul, Brasil, 2023 (N=864)

Método	Etapa I (30 dias)	Etapa II (30 dias)	Etapa III (30 dias)	Total de Avaliações
**Visual**	96	96	96	288
**ATP**	96	96	96	288
**UFC**	96	96	96	288
***Staphylococcus aureus* / MRSA**	96	96	96	288
**Total**	288	288	288	864

*ATP – trifosfato de adenosina; UFC – unidades formadoras de colônias; MRSA – Staphylococcus aureus resistente à meticilina.*

As duas instituições possuíam protocolos de limpeza concorrente e terminal, sendo que a limpeza terminal era realizada uma vez por semana ou quando um paciente recebia alta, era transferido ou ocorria um óbito. Já a limpeza concorrente era realizada uma vez ao dia. Os PE e os PSA eram os responsáveis pela LDS.

Entretanto, em ambas as UTIs, verificou-se que cada profissional utilizava um produto e um insumo diferentes para realizar a LDS, indicando a ausência de padronização antes da intervenção educativa realizada na etapa II do estudo. Não foi evidenciada a realização de capacitações específicas sobre a LDS em ambas as UTIs antes do estudo; os treinamentos eram restritos a temas relacionados à técnica de lavagem das mãos e ao uso das Precauções Padrão.

### Coleta e organização dos dados

Os métodos de monitoramento utilizados incluíram inspeção visual, ATP por bioluminescência, contagem de UFC e identificação de microrganismos patogênicos, como MRSA. As coletas foram realizadas pela pesquisadora imediatamente antes e 5 minutos após a conclusão da LDS da manhã ou da tarde. Esse procedimento visava garantir que as superfícies estivessem completamente secas para evitar possíveis interferências nas leituras devido ao contato com os produtos sanitizantes^(17)^.

O estudo foi composto de três etapas: I - diagnóstico situacional, II - intervenção educativa e monitoramento imediato, e III - monitoramento a curto prazo. Após a realização das avaliações nos hospitais, foi realizada uma intervenção educativa com a apresentação dos resultados e proposta de como realizar a LDS. A intervenção educativa foi inicialmente avaliada por 3 membros da Comissão de Controle de Infecção Hospitalar (CCIH) das duas instituições, sendo um médico infectologista e duas enfermeiras. Após essa validação pela CCIH, a intervenção foi coordenada com as equipes de enfermagem das UTIN e dos serviços ambientais.

Padronizou-se que a LDS seria realizada duas vezes por turno (no início e no final do turno). Considerando que, com o tempo, as superfícies voltam a ser colonizadas e os valores de ATP se elevam, a limpeza intermediária duas vezes ao dia apresentou benefícios em comparação com a realizada apenas uma vez ao dia^(18)^.

Padronizou-se também o uso de desinfetante hospitalar para as seis superfícies monitoradas, à base de cloreto de alquil dimetil benzil amônio, recomendado para a desinfecção de superfícies fixas e de artigos não críticos, devido à sua ação microbicida e atividade de amplo espectro contra bactérias, leveduras, fungos, vírus e esporos. O produto deve ser aplicado deixando a superfície umedecida por 10 minutos^(11)^. Também foi definido o uso de panos de microfibra como insumo para a realização da limpeza, considerando seu melhor desempenho na remoção microbiana^(19)^.

Para mensurar a matéria orgânica, o ATP por bioluminescência foi empregado com o auxílio de um luminômetro portátil (NGi 3M™ Clean-Trace™, St Paul, MN) e de um swab (3M™ Clean-Trace™ ATP Superfície). Superfícies com leitura menor que 250 RLU foram consideradas limpas^(20,21)^. A área de amostragem para a coleta do swab de ATP foi padronizada em 25 cm²^(22)^.

Para monitorar os microrganismos aeróbios totais, foram utilizadas placas de contato de 24 cm² do tipo *Rodac Plate* ^®^ (Biocen do Brasil), contendo ágar triptona de soja e neutralizantes. Após pressionar as placas sobre as superfícies por 10 segundos, estas foram incubadas a 37°C por 24 a 48 horas. A leitura das placas foi realizada com um contador de colônias digital (Logen LS6000; Texas Instruments Inc., Dallas, TX), considerando superfícies limpas aquelas com menos de 2,5 UFC/cm², ou seja, menos de 60 UFC para uma placa de 24 cm².

Além disso, foi analisada a presença de *Staphylococcus aureus* utilizando placas Petrifilm™ (3M™, St Paul, MN, USA), um sistema de meio de cultura pronto para uso, contendo um agente gelificante solúvel em água fria. O meio cromogênico modificado de Baird Parker na placa é seletivo e diferencial para *Staphylococcus aureus*. Colônias com coloração vermelho-violácea apresentaram resultado confirmado para *Staphylococcus aureus*. A inoculação ocorreu a 35°C por 24 a 48 horas. As colônias com coloração vermelho-violácea foram repicadas na placa Chromagar MRSA de tamanho 90 x 15 mm, que possui meio cromogênico destinado ao isolamento e diferenciação de MRSA. Após 48 horas, as placas que apresentaram mudança em sua coloração indicaram a presença de MRSA.

Para a avaliação visual das superfícies, foi realizada uma inspeção inicial para identificar áreas de sujeira, manchas ou qualquer tipo de resíduo visível. Utilizaram-se critérios pré-estabelecidos para avaliar a limpeza, observando a remoção de sujeira visível, poeira e outros contaminantes^(23)^.

### Análise dos dados

Os dados foram analisados utilizando os seguintes testes estatísticos: teste para duas proporções, para comparar a frequência de ocorrência de superfícies reprovadas entre os métodos de monitoramento (UFC, ATP e *Staphylococcus aureus*); correlação de Spearman, para observar possíveis correlações entre a quantificação de variáveis contínuas (ATP e contagem microbiana em cada superfície antes e após a limpeza e a desinfecção); e curva ROC, para verificar se o teste de ATP por bioluminescência é efetivo na determinação da qualidade da limpeza e desinfecção de uma superfície em relação ao padrão-ouro de avaliação microbiológica.

Todos os testes estatísticos foram aplicados com nível de significância de 5% (P < 0,05), e os softwares utilizados foram Minitab 17 (Minitab Inc.) e MedCalc 16.8 (MedCalc^®^).

## RESULTADOS

Ao final das três etapas, foram 864 avaliações incluindo ATP, inspeção visual, contagem de UFC e detecção de *Staphylococcus aureus* (SA) e a testagem de sua resistência. Os dados serão apresentados em UTIN A privada e UTIN B pública.

A Tabela 1 mostra os coeficientes de Spearman e os respectivos valores P referentes à correlação entre as metodologias empregadas no estudo para UTI A.

**Tabela 1:** Coeficiente de correlação de Spearman (Valor de *p*) entre trifosfato de adenosina (unidades relativas de luz) e bactérias aeróbias (unidades formadoras de colônia) nas amostras obtidas das superfícies – Unidade de Terapia Intensiva Neonatal A

Fases	Superfícies	ATP e UFC		ATP e SA		UFC e SA	
		r^1^	Valor de *p* ^2^	r	Valor de *p*	r	Valor de *p*
I	Incubadora	-0,098	0,818	0,252	0,547	0,000	1,000
	Mesa de preparo	0,139	0,742	0,427	0,291	0,263	0,529
	Bomba de infusão	-0,643	0,086	0,206	0,624	-0,289	0,488
	Balança	-0,012	0,978	0,412	0,310	0,415	0,307
	Poltrona	-0,171	0,686	-0,084	0,844	-0,049	0,908
	Balcão	0,587	0,126	0,179	0,672	0,754	0,031
II	Incubadora	0,922	0,001	0,234	0,577	0,486	0,222
	Mesa de preparo	0,299	0,471	-0,577	0,134	0,249	0,552
	Bomba de infusão	0,527	0,180	0,655	0,078	-0,094	0,825
	Balança	0,415	0,307	0,183	0,664	0,217	0,606
	Poltrona	0,311	0,453	0,430	0,288	0,117	0,782
	Balcão	-0,024	0,955	-0,192	0,650	0,217	0,606
III	Incubadora	-0,572	0,138	-	-	-	-
	Mesa de preparo	0,455	0,257	-	-	-	-
	Bomba de infusão	-0,275	0,509	0,083	0,845	0,412	0,310
	Balança	-0,482	0,227	-0,218	0,604	0,331	0,423
	Poltrona	0,530	0,177	-0,082	0,846	0,000	1,000
	Balcão	-0,610	0,108	-	-	-	-

*ATP – trifosfato de adenosina; UFC – unidades formadoras de colônia; AS – Staphylococcus aureus;*

1 *r: coeficiente de correlação de Spearman;*

2 *Valor de p referente ao teste de correlação de Spearman a p<0,05.*

Os resultados revelaram correlações específicas em diferentes superfícies e fases de avaliação. Na fase I, observou-se uma correlação significativa e positiva entre a quantificação de UFC e SA no balcão (P = 0,031), indicando que maiores valores de UFC estão associados a maiores valores de SA. Na fase II, identificou-se também uma correlação significativa e positiva entre a quantificação de ATP e UFC na incubadora (P = 0,001), sugerindo que, à medida que os valores de ATP aumentam, os valores de UFC também aumentam. Essas correlações estão apresentadas de forma visual na Figura 1.

**Figura 1:** Correlação entre bactérias aeróbias (unidades formadoras de colônia) e *Staphylococcus aureus* no balcão (fase I), e entre trifosfato de adenosina (unidades relativas de luz) e bactérias aeróbias na incubadora (fase II) – Unidade de Terapia Intensiva Neonatal A

Na Tabela 2 é possível verificar a aplicação do teste de correlação de Spearman com os dados da UTI B.

**Tabela 2:** Coeficiente de correlação de Spearman (Valor de *p*) entre trifosfato de adenosina (unidades relativas de luz) e bactérias aeróbias (unidades formadoras de colônia) nas amostras obtidas das superfícies – Unidade de Terapia Intensiva Neonatal B

Fases	Superfícies	ATP e UFC		ATP e SA		UFC e SA	
		r^1^	Valor de *p* ^2^	r	Valor de *p*	r	Valor de *p*
I	Incubadora	0,214	0,610	-0,247	0,555	0,289	0,488
	Mesa de preparo	0,482	0,227	-0,325	0,432	0,342	0,407
	Bomba de infusão	-0,719	0,045	0,701	0,053	-0,390	0,339
	Balança	0,548	0,160	-	-	-	-
	Poltrona	-0,036	0,932	0,342	0,408	-0,210	0,618
	Balcão	0,252	0,548	0,055	0,898	0,590	0,124
II	Incubadora	-0,575	0,136	0,038	0,929	0,147	0,729
	Mesa de preparo	0,190	0,651	0,507	0,199	0,114	0,788
	Bomba de infusão	0,168	0,691	-0,051	0,905	-0,223	0,595
	Balança	-0,310	0,456	-	-	-	-
	Poltrona	0,216	0,608	-	-	-	-
	Balcão	0,168	0,691	-	-	-	-
III	Incubadora	0,275	0,509	0,082	0,846	0,083	0,845
	Mesa de preparo	0,434	0,283	0,327	0,429	0,237	0,573
	Bomba de infusão	0,072	0,866	-	-	-	-
	Balança	0,262	0,531	-	-	-	-
	Poltrona	0,476	0,233	0,203	0,630	0,343	0,406
	Balcão	0,071	0,867	0,577	0,134	0,247	0,555

*ATP – trifosfato de adenosina; UFC – unidades formadoras de colônia; AS – Staphylococcus aureus;*

1 *r: coeficiente de correlação de Spearman;*

2 *Valor de p referente ao teste de correlação de Spearman a p<0,05.*

Os dados apresentados na Tabela 2 mostram uma correlação significativa entre a quantificação de ATP e UFC na bomba de infusão durante a fase I (P=0,045). Notavelmente, essa correlação é negativa, o que implica que o aumento da contagem microbiana está associado a uma diminuição no escore de ATP (Figura 2).

**Figura 2:** Correlação entre trifosfato de adenosina (unidades relativas de luz) e bactérias aeróbias (unidades formadoras de colônia) na bomba de infusão (fase I)

**Figura 3:** Curva de característica de operação do receptor (ROC) dos métodos de quantificação de trifosfato de adenosina em relação ao padrão ouro de contagem microbiana

A análise da curva ROC para os dados da UTIN A possibilitou a avaliação dos métodos de quantificação de ATP e inspeção visual em comparação com o padrão-ouro de contagem microbiana. A quantificação de ATP por bioluminescência apresentou uma sensibilidade de 75%, especificidade de 52,8%, valor preditivo positivo de 61,37% e valor preditivo negativo de 67,86%.

Com base na referência de CCA < 2,5 UFC/cm² para considerar uma superfície limpa, a análise ROC indica que superfícies com ATP abaixo de 28 URL podem ser consideradas aprovadas, sugerindo um critério eficaz para a avaliação da limpeza nas superfícies da UTIN A. Esse valor representa o ponto de maior especificidade e sensibilidade, conforme ilustrado na figura abaixo.

A curva ROC com os dados da UTIN B, ao avaliar o método de quantificação de ATP por bioluminescência em comparação com a contagem microbiana, revelou os seguintes resultados: sensibilidade de 100%, especificidade de 60,5%, valor preditivo positivo de 71,68% e valor preditivo negativo de 100%. Considerando a referência de CCA < 2,5 UFC/cm² para a definição de superfície limpa, a análise ROC sugere que superfícies com ATP menor ou igual a 38 URL podem ser consideradas aprovadas. Esse ponto de corte foi identificado como o de maior especificidade e sensibilidade, conforme ilustrado na Figura 4.

**Figura 4:** Curva de característica de operação do receptor (ROC) dos métodos de quantificação de trifosfato de adenosina em relação ao padrão ouro de contagem microbiana

## DISCUSSÃO

O estudo visou correlacionar os métodos de monitoramento da LDS em duas UTIN e comparar os valores de corte para o ATP. A análise revelou diferenças significativas na correlação entre os métodos de monitoramento e nos valores de corte deste primeiro teste, respectivamente, entre as duas unidades, apesar de utilizarem protocolos de desinfecção semelhantes e os mesmos produtos.

A correlação entre ATP, UFC e inspeção visual em superfícies de UTIN apresenta relevância clínica significativa para o controle de IRAS. A identificação de correlações positivas entre ATP e UFC em superfícies específicas sugere que o ATP pode ser uma ferramenta útil para avaliar a presença de matéria orgânica e, potencialmente, indicar a contaminação microbiológica. Entretanto, como a quantidade de ATP reflete matéria biológica total e não exclusivamente microrganismos, interpretações isoladas podem levar a erros na avaliação da eficácia da limpeza.

A inspeção visual, embora importante, mostrou limitações quando usada isoladamente, visto que superfícies visualmente limpas podem apresentar altas quantificações de ATP e UFC, indicando a presença de microrganismos e risco potencial de infecções. Dessa forma, a correlação entre essas medições evidencia a necessidade de uma abordagem multidimensional, associando diferentes métodos de monitoramento para garantir ambientes mais seguros para os pacientes, especialmente em UTIN, onde os recém-nascidos são altamente vulneráveis a infecções^(13,23-25)^.

Em relação à correlação dos métodos, observa-se que, mesmo sendo duas UTIN localizadas em uma mesma cidade, com protocolos de limpeza semelhantes, mesmo número de leitos e utilizando os mesmos produtos para a realização da LDS, apresentaram dados divergentes quanto à correlação dos métodos e ao valor de corte do ATP. Na UTIN A, foi verificada correlação positiva apenas entre a quantificação de UFC e *Staphylococcus aureus* no balcão na fase I (P = 0,031) e entre a quantificação de ATP e UFC na incubadora na fase II (P = 0,001). Já na UTIN B, foi verificada correlação entre a quantificação de ATP e UFC em apenas uma superfície, na bomba de infusão na fase I (P = 0,045).

Essa variação também foi observada em estudos realizados em diferentes cenários. Em enfermaria de internação pediátrica, foi identificada uma correlação significativa entre os métodos de medição de ATP e contagem microbiana nas superfícies da grade da cama (P = 0,009) e da poltrona (P = 0,018)^(16)^. Em estudo realizado em uma unidade de emergência (pronto-socorro), verificou-se também correlação significativa entre os métodos de quantificação de ATP e contagem microbiana para a maçaneta do banheiro feminino (P = 0,526; P = 0,008)^(26)^.

Em pesquisa realizada na atenção primária, obteve-se correlação significativa entre os métodos de quantificação de ATP e contagem microbiana aeróbia apenas para a maca do paciente (P = 0,001), apresentando um coeficiente de correlação de Spearman igual a r = 0,649^(27)^. Em outro estudo realizado em 10 superfícies hospitalares e de lar de idosos, verificou-se correlação fraca entre os valores de URL e UFC (R = 0,244; P < 0,001)^(28)^.

As variações nos resultados de correlação sofrem influência de diversos aspectos, entre eles, o perfil epidemiológico de colonização/contaminação ambiental e o fato de que a correlação entre ATP e UFC nem sempre será linear. Uma superfície com alta carga de matéria orgânica identificada pelo ATP não necessariamente apresentará alta carga de contaminação por microrganismos, conforme mensurado pelas UFC^(16)^. Nesse sentido, é importante destacar que o ATP mensura uma ampla variedade de material biológico, sendo as colônias apenas um dos diversos elementos que podem estar presentes na matéria orgânica. Assim, o ATP não permite apontar exclusivamente a carga microbiana existente na superfície^(22)^.

Ao comparar os parâmetros da curva ROC, verifica-se variação nos valores de sensibilidade, especificidade, valor preditivo positivo e valor preditivo negativo entre as duas UTIN. Essa variação nos valores encontrados em relação à sensibilidade e especificidade, comparando as duas UTIN, também é observada em outros estudos individuais realizados em diferentes cenários (atenção primária, ambulatório e unidade de emergência), que avaliaram a quantificação de ATP e a inspeção visual considerando a contagem microbiana como padrão-ouro na curva ROC^(12,13,23)^. Esses resultados destacam a importância de utilizar diferentes métodos para monitorar a LDS, devido às suas distintas sensibilidades e especificidades^(26)^.

No que tange ao valor de corte do ATP, na UTIN privada o valor foi inferior a 28 URL, enquanto na pública foi menor ou igual a 38 URL. Estudos anteriores identificaram diferentes valores de corte para o ATP, como 48 URL em atenção primária e 49 URL em atenção ambulatorial^(13,27)^. A variação nos valores obtidos pode estar relacionada à diversidade nos critérios de corte do ATP e à variação dos sistemas utilizados para monitorar o ATP. Além disso, falhas na remoção da sujidade podem favorecer a permanência do ATP por mais de 24 horas nas superfícies, mesmo após a morte dos microrganismos pela ação dos desinfetantes, gerando resultados elevados de ATP e baixos de UFC^(23)^. Ou seja, os microrganismos são apenas um dos componentes presentes na matéria orgânica^(28)^.

Seguir protocolos eficazes de limpeza e desinfecção, como o uso de produtos à base de alvejantes, desinfetantes de amônio quaternário e peróxido de hidrogênio líquido ou vaporizado, permite que os profissionais dos serviços ambientais eliminem ou reduzam a presença de patógenos nos ambientes de saúde. Isso é especialmente crucial para controlar a propagação de patógenos que representam uma séria ameaça à segurança dos pacientes^(5)^.

É pertinente apontar que diferentes tipos de aparelhos e marcas estão disponíveis para a mensuração do ATP, o que também contribui para a variabilidade dos resultados^(22)^. Outro aspecto relevante refere-se à área de amostragem utilizada para as coletas do swab de ATP. Se as leituras de ATP não forem calibradas de acordo com a área de amostragem, os dados obtidos podem resultar em interpretações complexas e de difícil comparação, principalmente em superfícies não uniformes^(22)^.

Em uma pesquisa realizada em três fases, os autores verificaram que os valores de ATP aumentaram cinco horas após a limpeza nas fases 1 e 2, e oito horas após a limpeza na fase 3, sendo que as enfermarias estruturalmente mais antigas apresentaram maior teor de ATP^(18)^. Embora o ATP não deva substituir os testes microbiológicos, ele permite fornecer feedback imediato aos profissionais em relação ao processo de LDS realizado^(29)^.

Com o objetivo de minimizar a variabilidade do ATP, o estudo abordou a amostragem duplicada por *swab*. Contudo, no mesmo estudo, foi identificado um percentual de apenas 10% de casos de falsos negativos, ou seja, situações em que a primeira amostra apresentou um resultado de aprovação (< 100 URL) e a segunda coleta foi reprovada (> 100 URL)^(30)^.

### Limitações do estudo

A análise da curva ROC referente à quantificação de *Staphylococcus aureus* não pôde ser realizada devido à falta de representatividade amostral e à alta frequência de valores nulos. Embora o efeito Hawthorne, que pode ocorrer quando os profissionais alteram seu comportamento ao saber que estão sendo observados, fosse uma possibilidade, ele foi minimizado. Na fase 1, os profissionais não foram informados sobre os objetivos do estudo, e nas fases seguintes, as coletas foram realizadas antes da chegada dos PSA ao setor, garantindo que as práticas de LDS na UTIN não fossem influenciadas pela observação.

Vale destacar que a ausência de padronização nos produtos de limpeza e a falta de capacitação das equipes responsáveis pela LDS são pontos críticos, podendo gerar variações nos resultados e aumentar a chance de viés, uma vez que a técnica de cada profissional pode influenciar na efetividade da LDS.

### Contribuições para a Área

Embora se reconheça a importância do uso de métodos de monitoramento da LDS, ainda se observa que, na maioria dos serviços, a inspeção visual é utilizada predominantemente, com enfoque nos aspectos estéticos. Este estudo reforça a importância de incorporar outros métodos de avaliação. Ademais, ele preenche uma lacuna na literatura sobre a eficácia dos métodos de monitoramento em UTIs neonatais, enfatizando a relevância de uma abordagem multifacetada e adaptada às especificidades de cada ambiente ao estabelecer padrões de LDS.

Implementar intervenções educativas e padronizar procedimentos pode melhorar a eficácia da desinfecção e, consequentemente, reduzir o risco de infecções hospitalares, garantindo um ambiente mais seguro para os pacientes neonatais.

## CONCLUSÕES

Este estudo revelou variações significativas na correlação dos métodos de monitoramento da LDS nas duas UTIN analisadas, apesar de ambas estarem localizadas na mesma cidade, possuírem o mesmo número de leitos e utilizarem protocolos e produtos de desinfecção semelhantes. Na UTIN A, foram observadas correlações positivas entre UFC e *Staphylococcus aureus,* e entre ATP e UFC, enquanto, na UTIN B, não foram identificadas correlações positivas entre os métodos. Além disso, os valores de corte do ATP variaram entre as UTIN, indicando diferentes níveis de eficácia na desinfecção.

Esses achados apontam para a necessidade de abordagens personalizadas no monitoramento da limpeza e desinfecção, considerando as particularidades de cada unidade. A divergência nos resultados sugere que fatores específicos de cada UTIN, como práticas de limpeza, características das superfícies e comportamento dos profissionais de saúde, podem influenciar a eficácia dos métodos de desinfecção.

A variação nos valores de corte do ATP também indica a importância de definir padrões específicos para cada contexto, em vez de aplicar um valor universal. Nesse sentido, recomenda-se a adaptação dos protocolos de monitoramento da limpeza, levando em consideração as especificidades de cada unidade, e o ajuste dos valores de corte do ATP conforme as características locais, a fim de otimizar a eficácia dos processos de desinfecção e garantir a segurança dos pacientes.

## Data Availability

Não se aplica.
